# Supplementary material for: The multi-faceted effects of technology-driven productivity surge in the crop & livestock sector in Greece: Evidence from the FABLE Calculator
Source: PLoS One. 2026 Feb 10;21(2):e0341900. doi: 10.1371/journal.pone.0341900 (PMC12890123; doi:10.1371/journal.pone.0341900)
Supplement: S1 Appendix — Tables A1 and A2 present the description of the distinct pathways for the Greek agri-food sector, as documented in the FABLE 2023 Scenathon. (DOCX) [file pone.0341900.s002.docx]

# **Appendix I**

**Greece Current Trends and National Commitments**

Tables A1 and A2 present the description of the distinct pathways for the Greek agri-food sector, as documented in the [FABLE 2023 Scenathon](https://fableconsortium.org/content/pages/scenathon-2023/).

Table A1: Pathway Narratives

| **CURRENT TRENDS** |  | **NATIONAL COMMITMENTS** |
| --- | --- | --- |
| We do not act differently than the past decade / today. |  | National actions/policies are aligned with national commitments |
| The Current Trends Pathway projects key elements of the food, land-use, energy, and biodiversity systems conditional on no significant policy and behavioral changes in Greece for the 2020-2030 period. The continuation of business as usual implies high urbanization and an uptick in economic activity, no change in dietary consumption for the general population, 50% surge in key exports and increased reliance on food imports. Moreover, we assume no substantial shift in biofuel demand, no afforestation target and no change in post-harvest losses. This Pathway is embedded in a global GHG concentration trajectory that would lead to a radiative forcing level of 6 W/m2 (RCP 6.0), or a global mean warming increase likely 2-3°C above pre-industrial levels. |  | Under the National Commitments Pathway, we underscore specific numerical and qualitative targets based on Greece's NECP, the Pissarides Committee Plan for the Greek Economy and the commitments accruing from EU participation. The pathway entails medium to high speed of economic growth, shift to a healthy diet (as described by the Lancet Committee), and reduced imports. Nonetheless, exports are expected to double by 2050 reflecting the country’s aspiration for outward-oriented economic growth and productivity is expected to surge both for crops and for livestock production. This Pathway is embedded in a global GHG concentration trajectory that would lead to a lower radiative forcing level (RCP 4.5) and assumes expansion of protected areas and an increase in agricultural land under organic practices. |

Table A2: Pathway Assumptions

| **TOPIC** | **INDICATOR** | **CURRENT TRENDS** | | **NATIONAL COMMITMENTS** | |
| --- | --- | --- | --- | --- | --- |
| **1. Macroeconomics** | 1.1) GDP per capita | 2% y-o-y Growth | | 3.5% y-o-y Growth until 2030 | |
|  | 1.2) Population | 1.5 - 2.5 million reduction by 2050 | | Maintain population no less than 10 million | |
|  | 1.3) Inflation | 4.2% in 2023 2.4% in 2024 | | 2% y-o-y | |
|  |  |  |  |  |  |
|  | 1.4) Inequalities | Gini Index 33.5 in 2020 - moving away from Target according to SDG 10 | | Gini Index drop below 30 by 2030 | |
| **2. Land** | 2.1) Constraints on agricultural expansion/deforestation | Promotion of no deforestation and expansion of agricultural land tied to agroforestry targets | | Increase Legislation Stringency regarding deforestation for agricultural expansion and enhance monitoring and implementation | |
|  | 2.2) Afforestation, and forest plantations targets | 3.5 - 4 Mha of Forest Area in 2030. | | 4.2 - 4.5 Mha of Forest Area in 2030. | |
|  |  |  |  |  |  |
|  |  |  |  |  |  |
|  | 2.3) Urban and settlements area | 128,900 sq. km. as of 2015 urban land area | | 0.2% growth | |
|  | 2.4) Protected areas | 1249 protected areas, 30.2% of land, 19.4% of sea | | By 2030, protected areas cover at least 30% of the land area and sea of the country | |
|  |  |  |  |  |  |
|  |  |  |  |  |  |
| **3. Productivity and management** | 3.1) Crop productivity for the key crops | | As of 2022, agricultural productivity: Sugar beet (excluding seed): 42.91 tonne/ha; Potatoes (including seed potatoes): 26.45 tonne/ha; Grain maize and corn-cob-mix: 19.75 tonne/ha; Rice: 5.74 tonne/ha; Wheat and spelt: 2.72 tonne/ha; Barley: 2.44 tonne/ha Rye and winter cereal mixtures (maslin): 1.76 tonne/ha; Tobacco: 1.43 tonne/ha; Cotton fibre: 1.29 tonne/ha; Oats: 1.18 tonne/ha | | Converge to EU average in crop yields for main crops: Cereals, Rice, Olives, Citrus fruits, Nuts. Green maize: 3.5 tn/ha, Wheat & Spelt: 4 tn/ha; Barley: 4 tn/ha, Cotton Fibre 1.5 tn/ha, Oats 3.5 tn/ha, Rye: 3.5 tn/ha |
|  | 3.2) Cropland under agroecological practices | Area under organic farming as of 2020, 10.2% of utilized agricultural area | | 20% or above by 2050 | |
|  | 3.3) Livestock productivity for the key livestock products | As of 2019, livestock production: | | >200 Hen Eggs and double the yield for Cattle Milk, Goat Milk, Pig Meat and Goat & Sheep Meat | |
|  |  | Bovine: 231.8 Kg/ head | |  |  |
|  |  | Sheep and goat: 11.3 Kg/ head | |  |  |
|  |  | Pig: 67.7 Kg/ head | |  |  |
|  |  | Chicken Meat: 1.7 Kg/head | |  |  |
|  |  | Cattle Milk: 7.6 Kg/head | |  |  |
|  |  | Goat Milk: 1.4 Kg/head | |  |  |
|  |  | Hen Eggs: 180 Eggs/head | |  |  |
|  | 3.4) Pasture stocking rate | Minimum stocking density levels for pastureland (which are set at 0.2 LU/ha for all categories of animal) | | Maintain stocking rate around current levels | |
|  | 3.5) Forest management | Permanent deforestation halted. Forests in continual deterioration due to poor management, competitive agricultural and settlement uses, intense pasture and summer fires. | | By 2030, promote the implementation of sustainable management of all types of forests, halt all deforestation, increase thinning and pruning as preventive measures and increase forest sector contribution to GDP from 0.05% to the EU average of 0.2% | |
|  |  | High slopes make harvesting extremely difficult, occurring only during May – Sept. when climatic conditions are favorable but this is an inappropriate period. | |  |  |
| **4. Trade** | 4.1) Share of consumption which is imported for key imported products (%) | 8.5 - 10 billion yearly imports of agricultural products | | Reduction in order to achieve neutral agricultural trade balance | |
|  | 4.2) Evolution of exports for key exported products (1000 tons) | 6 - 7 billion yearly agricultural exports 2010 - 2020 - 9~10% of total exports (goods and services) | | Increase in order to achieve neutral agricultural trade balance | |
| **5. Food** | 5.1) Average dietary composition | Average dietary energy supply, 2019-2021: 3412 kcal / capita/ day, of which, as per 2019: cereals: 811 kcal / capita/ day; fats and oils: 842 kcal / capita/ day; meat: 290 kcal / capita/ day; sugar: 332 kcal / capita/ day; roots, tubers and pulses: 118 kcal / capita/ day; fruit and vegetables: 287 kcal / capita/ day; dairy and eggs (exl. butter) 430 kcal / capita/ day; beverages and other: 189 kcal / capita/ day; fish and seafood: 38 kcal / capita/ day. | | Diet shifts to the Lancet diet by 2050 (EAT, Planetary Health diet) | |
|  | 5.2) Share of food consumption which is wasted at household level | As per 2019, the total per capita food waste generation in Greece is estimated to be 76.1 kg/inh-y. | | Reduce food waste by 30% by 2035 and to relative EU median levels by 2050 | |
| **6. Biofuels** | 6.1) Targets on biofuel and/or other bioenergy use | The projection of 2030 predicts that the bioethanol share will fall to 71%, the biodiesel will also fall to 12%, but BTL (biomass to liquids) will emerge and stand to 12% market share, especially due to second and third generation biofuels. | | Greece plans to increase the RES-T to 19% in 2030 (10% without multipliers) with biofuels accounting for 80% of the RES-T or about 371 ktoe (vs. 157 ktoe in 2018). Contribution from biofuels from Annex IX-A feedstocks is expected to reach 197 ktoe in 2030 (vs. 0 ktoe in 2018). Greece has introduced a target for advanced biofuels of 0.2% in volume. | |
|  |  |  |  |  |  |
|  | 6.2) Targets on other non-food use | - | | The contribution made by biofuels produced from wastes, residues, non-food cellulosic material, and ligno-cellulosic material shall be twice that made by other biofuels | |
|  |  |  |  |  |  |
|  |  |  |  |  |  |
| **7. Water** | 7.1) Irrigated crop area | Over the period 2011 - 2018, 36% of the agricultural area was irrigated. | | Greece's target is to improve water management on 17.5% of agricultural land and water efficiency for 5% of irrigated land through irrigation infrastructure | |
|  |  |  |  |  |  |
