# Supplementary material for: The multi-faceted effects of technology-driven productivity surge in the crop & livestock sector in Greece: Evidence from the FABLE Calculator
Source: PLoS One. 2026 Feb 10;21(2):e0341900. doi: 10.1371/journal.pone.0341900 (PMC12890123; doi:10.1371/journal.pone.0341900)
Supplement: Attachment — Submitted filename: Reviewer PLOS ONE.docx [file pone.0341900.s003.docx]

Reviewer’s Comments: The multi-faceted effects of technology-driven productivity surge in the crop and livestock sector in Greece: Evidence from the FABLE Calculator

The authors use the FABLE Calculator to assess the intricate relationship between improved productivity in crops and livestock on environment, showing that enhancing productivity reduced GHG emissions by up to 29% until 2030 and 62% by 2050. The authors compare BAU and Enhanced Productivity Pathways, taking into account policy documents. The authors provide evidence for the use of precision agriculture for increasing agricultural productivity in Greece. Moreover, horizontal and vertical policies for the development, adoption and dissemination of latest technologies are proposed.

**General comments:**

The paper elaborates on the use of technology-driven productivity surge in crop and livestock in Greece, using the FABLE calculator. However, the dataset summary is missing in the text. The reader therefore cannot make sense of what data was used for the analysis in the FABLE calculator.

**Specific comments:**

In the Abstract, the figures for reduction of GHG emissions are 21% until 2030 and 52 % until 2050. However, in the text, the values are 29% and 62% respectively. The authors must correct the values used.

The abstract states that the study demonstrates the promotion of biodiversity conservation.

Line 81. References to be inserted in brackets.

Lines 87, 89, 92. Research questions should end with question marks.

The figures in the papers are in reverse order with Figure 1 appearing as the last figure.

Line 249. Period missing between costs and Total costs.

Line 275 meat consumption is expected to fall and not deteriorate.

Lines 352-354 are repeated in line 359-361.

Line 400 is incomplete.

Line 424 corroborate should be followed by ‘with’.
